# The effects of oral hydrolytic enzymes and flavonoids on inflammatory markers and coagulation after marathon running: study protocol for a randomized, double-blind, placebo-controlled trial

**DOI:** 10.1186/2052-1847-6-8

**Published:** 2014-02-22

**Authors:** Viola Grabs, David C Nieman, Bernhard Haller, Martin Halle, Johannes Scherr

**Affiliations:** 1 Department of Prevention, Rehabilitation and Sports Medicine, Klinikum rechts der Isar, Technische Universitaet Muenchen, Munich, Germany; 2 Human Performance Laboratory, Appalachian State University, North Carolina Research Campus, Kannapolis, NC, USA; 3 Institute for Medical Statistics and Epidemiology, Klinikum rechts der Isar, Technische Universitaet Muenchen, Munich, Germany; 4 DZHK (German Centre for Cardiovascular Research), partner site Munich Heart Alliance, Munich, Germany

**Keywords:** Exercise, Inflammation, Upper respiratory tract illness, Oral hydrolytic enzymes, Flavonoids

## Abstract

**Background:**

Regular moderate intensity physical activity positively influences the immune system with a lower incidence of upper respiratory tract infections (URTI) and lower levels of pro-inflammatory markers. However, marathon running due to its strenuous and prolonged nature results in immune perturbations with a major increase in pro-inflammatory markers and subsequent increased incidence of URTI. Furthermore, marathon running results in muscle damage and changes in hemostasis that promote a pro-thrombotic state.

Naturally occurring hydrolytic enzymes and flavonoids have antioxidant, anti-inflammatory and fibrinolytic effects, and may serve as countermeasures to exercise-induced inflammation, immune dysfunction and URTI.

The aim of this study is to determine whether the ingestion of oral hydrolytic enzymes and flavonoids before and after a marathon attenuates post-race muscle damage and inflammation, counters pro-thrombotic changes in hemostasis and decreases URTI incidence.

**Methods/design:**

The Enzy-MagIC-study (***Enzy*** mes, ***Ma*** rathon runnin ***G***, ***I*** nflammation, ***C*** oagulation) is a randomized, double-blind, placebo-controlled, monocenter phase I trial. 160 healthy males (age 20-65 years) will be randomized to receive either placebo or treatment (Wobenzym, MUCOS Pharma, Berlin, Germany) which contains the hydrolytic enzymes (bromelain, trypsin) and the flavonoid rutoside. One week before the marathon race, participants will begin daily ingestion of the investigational product (3×4 tablets). Intake will be continued for two weeks after the race (3×2 tablets per day). Clinical and laboratory measures will be collected 5-weeks and 1-week before the race, and immediately-, 24-h, 72-h, and 2 weeks after the race. The primary endpoint is the influence of the treatment on the pre-to-post marathon race plasma concentration change of the inflammatory marker interleukin-6 (IL-6). Secondary endpoints include the effect of treatment on salivary IgA concentration and the frequency of upper respiratory tract infections (URTI) for two weeks post-marathon as determined by the Wisconsin Upper Respiratory Symptom Survey (WURSS-24). Furthermore, changes of muscular and rheological parameters will be measured before and after the marathon race.

**Discussion:**

We hypothesize that marathon-induced inflammatory perturbations and the incidence of subsequent URTI, muscular damage, and changes of hemostasis can be positively influenced by the anti-edematous, anti-inflammatory, antioxidant, and fibrinolytic effects of oral hydrolytic enzymes and flavonoids (Wobenzym).

**Trial registration:**

ClinicalTrials.gov Identifier: NCT01916408

## Background

Regular moderate intensity physical activity has been shown to positively influence the immune system [1,2]. Clinically this is represented by decreased incidence of upper respiratory tract infections (URTI) and pro-inflammatory markers in individuals who participate in regular moderate-intensity physical activity [2,3]. In contrast, most studies indicate that URTI rates increase during the 1-3 week period following marathon-type race events due to transient alterations in immunosurveillance [4-6]. Strenuous and prolonged exercise such as marathon running results in a major increase in inflammatory markers (e.g. C-reactive protein, interleukin 6) [6-8]. IL-6 is a cytokine with a wide range of biological effects, including pro-inflammatory influences during sepsis [9]. IL-6 is a central mediator of the acute-phase response and primary determinant of hepatic production of C-reactive protein (CRP) [10]. Elevated levels of IL-6 and CRP have been found in low-grade systemic inflammation such as atherosclerosis and diabetes mellitus [11,12]. In healthy men an elevated IL-6 plasma concentration has been associated with increased vascular risk and myocardial infarction [13]. IL-6 may stimulate blood coagulation and has been suggested to be an independent predictor for sudden death [14,15].

However, it is discussed equivocally whether IL-6 is primarily a pro- or anti-inflammatory cytokine. IL-6 activity appears to be essential for both the effective management of acute inflammation and to balance pro- and anti-inflammatory activity [16]. Post-exercise increases in plasma IL-6 are related to exercise workloads, with the highest levels found after strenuous and prolonged exercise [6-8]. Plasma IL-6 concentration decreases rapidly after strenuous exercise and is followed by increased levels of anti-inflammatory cytokines such as IL-1ra and IL-10 inducing an anti-inflammatory environment [2,17,18]. Chronic exercise, especially when accompanied with weight loss, is associated with decreased CRP and IL-6. This is linked to a reduced atherosclerosis and cardiovascular disease incidence [11,12].

Besides immune alterations, marathon running results in muscular damage which causes elevated levels of muscle enzymes (e.g. creatine kinase) and muscle soreness [19]. Additionally, changes of hemostasis leading to a pro-thrombotic state can be seen after a marathon race [20].

Consumption of plant derived foods such as vegetables and fruits has been linked to a reduced incidence of chronic diseases such as cancer and atherosclerosis [21,22]. The protective effects of these foods are attributed to hydrolytic enzymes and flavonoids that have antioxidant, anti-inflammatory, and anti-microbial effects [23-25]. The most important and therefore most extensive investigated flavonoids are rutosid (synonym rutin) and its glycosyl quercetin. Both flavonoids are mostly found in plants (such as apples) [26]. There are several studies investigating the antioxidant properties of these two flavonoids [26-30]. Whereas bromelain is a protein-digesting enzyme found in pineapple [31]. Nutritional supplements are being investigated as countermeasures to exercise-induced inflammation, immune dysfunction, and URTI [32]. Polyphenol-rich supplements under certain exercise conditions have been shown to exert anti-inflammatory and antioxidant effects [6,27,28].

Supplementation with oral hydrolytic enzymes has been reported to have analgetic, anti-edematous and anti-inflammatory influences [33,34]. Bromelain has the potential to decrease neutrophil migration and secretion of pro-inflammatory cytokines [25,35]. Furthermore, Bromelain reduces platelet aggregation and exhibits fibrinolytic activities in dissolving fibrin clots [31]. Trypsin has antioxidant effects, influences the activation of protease-activated receptor 2, and decreases the inflammatory response in animal models and in studies with allergic respiratory disease [36-38].

Wobenzym (brand name: Wobenzym® plus, Phlogenzym® or Wobenzym PS® depending on the country) is an orally applied formulation composed of hydrolytic enzymes (bromelain, trypsin) and the flavonoid rutoside. Clinical trials in both humans and animals suggest a beneficial effect of this combination on inflammatory diseases. These include immunologically mediated atherosclerosis in rat aortic allografts and rheumatologic disease [39,40]. Following injury, Wobenzym has been shown to restore microcirculation, decrease pain, reduce inflammation and swelling, and alleviate musculoskeletal symptoms to the same extent as nonsteroidal anti-inflammatory drugs [34,36].

Taken together, supplementation with polyphenols and hydrolytic enzymes appears to be a promising approach to mitigate marathon-induced changes in inflammation, muscular damage and soreness, and rheological status.

Therefore, the aim of the study is to investigate the influence of hydrolytic enzyme and flavonoid supplementation on changes in inflammatory, muscular, and rheological status in healthy males participating in a marathon race.

## Methods

### Design and participants

**Figure 1:**
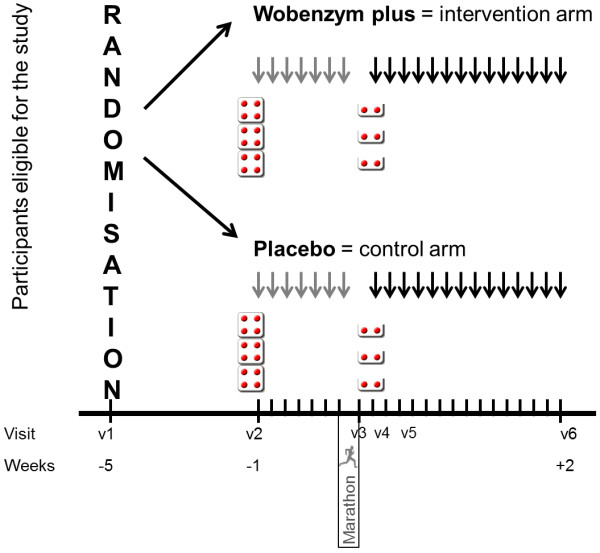
**Study design and treatment schema of Enzy-MagIC-study (*Enzy*mes,*Ma*rathon runnin*G*, *I*nflammation,*C*oagulation-Study).** V1: visit 1 five weeks before marathon race. V2: visit 2 one week before race. V3: visit 3 immediately after race. V4: visit 4 one day after race. V5: visit 5 three days after race. V6: visit 6 two weeks after race. Red dots: study medication. One week before the marathon race, participants will begin daily ingestion of the study medication (3×4 tablets), with intake continued for two weeks after the race (3×2 tablets per day).

Enzy-MagIC is a randomized, double-blind, placebo-controlled, monocenter phase I trial. Participants will be randomized in a 1:1 manner to receive either Wobenzym (treatment) or placebo. An overview of timing of randomization, treatment schema and data collection is presented in Figure 1.

A total of 160 healthy male athletes between the ages of 20 and 65 years who intend to participate in the Munich Marathon 2013 will be recruited from August-September 2013. The inclusion and exclusion criteria are presented in Table 1.

**Table 1:** In- and exclusion criteria for participants of the Enzy-MagIC-study

**Inclusion criteria**	**Exclusion criteria**
• Healthy* male	• Known cardiac disease
• Age 20-65 years	• Known allergy against the active ingredient of the study medication or pineapple, papaya, or kiwi
• History of at least one successfully finished half marathon	
• Intention to participate at the Munich Marathon 2013	• Known severe coagulopathy
• Subject is able to read, understand, and sign a written informed consent to participate in the Enzy-MagIC-study	• Known lactose intolerance
	• Pharmaceutical treatment for diabetes mellitus or arterial hypertension
	• Acute or chronic renal failure
	• Acute or chronic liver disease
	• Acute or chronic infection or inflammatory disease
	• Use of medications or supplements influencing immune function
	• Musculoskeletal or psychiatric disease
	• Neoplasia
	• Participation in other interventional trials

*Healthy means in accordance with the current WHO-definition a state of complex physical and mental well-being [41].

The study will be conducted in accordance with Good Clinical Practice guidelines, the guiding principles of the Declaration of Helsinki 2008, and local laws and regulations. The study protocol has been approved by the ethics committee of the University Hospital Klinikum rechts der Isar, Munich, Germany (approval reference number, 5820/13) and the Bundesinstitut für Arzneimittel und Medizinprodukte (BfArM), Bonn, Germany (Vorlagenummer, 4039219).

All participants will have to provide written informed consent. The trial is registered at ClinicalTrials.gov (NCT01916408).

### Interventions

One week before the marathon race, the participants will begin daily ingestion (oral use) of the investigational product Wobenzym (3×4 tablets) and for the two weeks after the marathon race 3×2 tablets. The treatment schema can be seen in Figure 1. Both Wobenzym dosages prior and after the race are in accordance with recommendations in the patient information sheet. In case of heavy inflammatory illnesses or after acute trauma, the dosage can be increased up to 12 tablets per day. Therefore, 12 tablets per day prior to the race should serve as a loading dose before the marathon race representing a inflammatory stimulus. After this loading period, the dosage is decreased to a maintenance dosage of 6 tablets per day. Based on this dosage regime, an adequate plasma concentration of rutoside, trypsin and bromelain can be expected in accordance to the study of Roots et al. [42].

The study product is Wobenzym, an orally applied enzyme-rutoside combination supplement. Each tablet contains 90 mg bromelain, 48 mg trypsin, and 100 mg rutoside. The current indications of the investigational product are swelling and inflammation caused by injuries/trauma, thrombophlebitis and inflammation of joints due to osteoarthritis. Pharmacological investigations in animals have proven no toxic, teratogenic or mutagenic characteristics after single, multiple or long-term intake.

Wobenzym (formerly known as Phlogenzym®) has been on the German market since 1991. Typical adverse reactions of this oral enzyme therapy are a change of consistency, color and odor of the stool. At higher doses, sensations of fullness, flatulence and diarrhea may appear. These symptoms may also be related to the excipient lactose. Very few allergic reactions have been reported. All reported adverse reactions have been minor and disappeared after discontinuation. None of the cases required further treatment. The placebo supplement for the control group consists of lactose-monohydrate and has no active ingredient.

### Laboratory measurements

Blood samples will be collected 5-weeks (visit 1) and 1-week (visit 2) before the race, and immediately (visit 3), 24-h (visit 4), 72-h (visit 5) and 2 weeks (visit 6) after the race. At these visits inflammation measures (IL-6, IL-1-β, IL-10, myeloperoxidase, high-sensitivity C-reactive protein (hs-CRP), leukocyte count) will be analyzed.

Muscle damage parameters (creatine kinase (CK), myoglobin, glutamate oxalacetate (GOT), lactate dehydrogenase (LDH)) will be collected and measured at each visit (1-6). The short form of the McGill-questionnaire will be used to assess muscular strain [43].

Rheological parameters (D-dimer, tissue plasminogen activator (tPA), platelet aggregation) will be collected and measured at each visit (1-6).

### Clinical measurements

Baseline data of the participants will be collected at the first visit. This includes questionnaires assessing training history, history of cardiovascular risk factors, physical examination, anthropometry, clinical chemistry, resting ECG, and echocardiography. One week prior to the race URTI-rates will be assessed daily by the Wisconsin Upper Respiratory Symptom Survey (WURSS-24) and will be compared between treatment and placebo group during the 2-week period following the race. The WURSS-24 is a reliable and valid tool for assessing symptoms, functional impairments, and global severity and global change of common cold [44]. Only complete questionnaires will be used for analyses.

Participants will be requested not to use medications influencing inflammation such as non-steroidal anti-inflammatory drugs. Furthermore, subjects will be asked to refrain from fatty food and from all polyphenol-containing foods, especially beverages such as wine, beer, and fruit juice, as well as fresh and dried fruits or vegetables. Nutritional intake will be recorded with a 3-days nutritional record before visit 2 and before the marathon race.

### Endpoints

#### Primary endpoint

The primary endpoint criterion is the effect of Wobenzym compared to placebo on plasma IL-6 concentration changes following the marathon race.

#### Secondary endpoints

Secondary endpoints include the effect of consuming Wobenzym on:

1. the frequency of upper respiratory tract infections (URTI) during the 2-week post-marathon period as determined by the Wisconsin Upper Respiratory Symptom Survey (WURSS-24).

2. pre-to-post marathon race changes in salivary IgA concentration.

3. pre-to-post marathon race changes of muscular (e.g. CK, myoglobin, GOT, LDH) and rheological (e.g. D-Dimer, tPA, platelet aggregation) parameters, and muscular strain ( McGill Pain Questionnaire).

4. pre-to-post marathon race changes of cytokine concentrations (e.g. IL-1-β, IL-10), oxidative markers (e.g. myeloperoxidase) and acute-phase-proteins (e.g. high-sensitivity C-reactive protein [hs-CRP]) as well as differential blood and leukocyte count (e.g. leukocytosis).

### Sample size

Sample size calculation was based on the primary endpoint criterion, the difference in marathon-induced change of plasma IL-6 between the study groups. Due to the expected right-skewed distribution of IL-6, differences in changes of logarithmized IL-6 levels (relative group changes) were considered as the base for inferential statistics. From previous publications a coefficient of variation (CV) of 0.60 for IL-6 changes was expected for the proposed study population [45]. Assuming a true relative difference of at least 25% between the groups, a sample size of 80 individuals per group is necessary to detect a difference in IL-6 changes between both groups (given the CV of 0.60) with a power of 90% on a two-sided level of significance of 0.05.

### Randomization

Participants will be randomized to Wobenzym or placebo in a 1:1 ratio using a pre-generated randomization list with variable block sizes.

### Blinding

According to the randomization list the IMP (either Wobenzym or placebo) will be labelled with the participant’s ID by the manufacturer MUCOS GmbH and will be sent to the principal investigator.

### Participants safety

#### Adverse events

Adverse events are undesirable signs or symptoms that occur during the study and may or may not be causally related to the treatment. All adverse events considered at any level to be related to the IMP will be recorded on eCRFs. A common cold or URTI episode is not regarded as an AE in this study.

#### Serious adverse events

Serious adverse events (SAEs) are defined as events that are fatal, life-threatening, disabling, incapacitating, or resulting in hospitalization, prolonged hospital stay, or malformation. All will be recorded in the eCRF, whether they are related to the IMP or not. According to preclinical data Wobenzym is a safe supplement. Any SAE considered at any level to be related to the IMP will be regarded as unexpected. All SAEs will be reported according to the GCP-guidelines.

#### Discontinuation

Participants have the right to withdraw from the clinical trial at any time and in any case without giving a reason. The investigator has the right to withdraw a subject for any reason which is in the best interests of the subject, including intercurrent illness or adverse events. Discontinued participants will not be replaced.

### Statistical methods

The difference of logarithmized IL-6 values will be calculated for each participant as distribution of IL-6 differences within the groups is expected to be skewed. Differences of logarithmized values will be compared between both study groups using a two-sample t-test. A two-sided level of significance of 5% will be considered for this primary efficacy analysis. Quantitative secondary endpoints will be analyzed using ANOVA methods, and qualitative data using chi-squared tests. Analysis of the primary endpoint and the secondary efficacy endpoints will be performed following the intention-to-treat (ITT) principle. These endpoints will also be assessed in the per-protocol dataset in the sense of sensitivity analyses.

The ITT dataset will consist of all trial subjects enrolled into the trial and randomized. For subjects with missing post marathon inflammation markers, a conservative replacement of the missing values will be conducted by imputing the lowest observed post marathon value for the placebo group and the highest observed value for the treatment group. The per-protocol dataset includes all trial subjects who were treated according to protocol. A tertiary dataset for analysis is the safety population. This population includes all trial subjects who received any IMP or other trial treatment.

### Data management

The trial database will be based on the MARVIN system, an electronic data base developed for clinical trials. The system is CDISC certified, well defined and represents a worldwide accepted data and documentation standard. This approach allows online plausibility checks and automated queries, and contains automatic validation rules. Every modification will be automatically tracked during the trial.The database is integrated in an IT infrastructure and safety system containing a backup and firewall system. The data are saved on a daily basis.The medical data in this trial are to be recorded in eCRFs. After completing the data input (including data cleaning) the database will be locked and can be exported for statistical analysis in SPSS or SAS format.

## Discussion

The Enzy-MagIC trial will investigate the effects of oral hydrolytic enzymes and flavonoids (Wobenzym) on marathon-induced changes in inflammation, muscle impairment, and rheological status in healthy males. Previous studies indicate that Wobenzym has anti-edematous, anti-inflammatory, antioxidant, and fibrinolytic effects [34,39,40]. Thus the hypothesis for this study is that Wobenzym supplementation for one week before and two weeks after participation in a competitive marathon race will mitigate inflammation and muscle damage, lower URTI incidence, and attenuate pro-thrombotic hemostatic changes.

Plasma IL-6 concentration is increased after strenuous prolonged exercise such as marathon running [6-9]. Polyphenols and trypsin may decrease IL-6 release [29,46,47] whereas bromelain may alter the release of other pro-inflammatory factors [35]. Thus a primary objective of this study is to investigate the effect of a mixture of rutoside, trypsin, and bromelain on marathon-induced changes in plasma IL-6. If proven to be efficacious in this setting, Wobenzym may emerge as a suitable alternative to NSAIDs.

Supplementation of a mixture of flavonoids has been reported to have anti-inflammatory and antioxidant effects after exercise [27,28]. Results from a randomized double-blind study with human marathon athletes ingesting a polyphenol-rich beverage for 3 weeks before and 2 weeks after marathon race showed a reduced incidence of URTI [6]. The athletes in this study drank 1-1.5 liters of non-alcoholic beer per day, making this approach very challenging. The use of Wobenzym tablets containing hydrolytic enzymes and flavonoids simplifies the supplementation process and should improve compliance.

Supplementation with bromelain in children with sinusitis showed a significantly faster recovery from symptoms compared to standard therapy [33]. Therefore the use of supplements with a mixture of hydrolytic enzymes and flavonoids might be a promising approach to attenuate marathon-induced immune dysfunction and lower URTI incidence and symptom severity.

Marathon running induces significant muscle soreness and damage, with large increases in creatine kinase and myoglobin [19]. Muscular damage and glycogen depletion have been associated in other studies with post-exercise increases in IL-6 and other inflammatory parameters [7]. Therefore, attenuation of muscle soreness and damage through use of Wobenzym should result in lower post-race plasma levels of IL-6. Other studies indicate that following injury, Wobenzym decreases pain and swelling, and is as effective as the nonsteroidal anti-inflammatory drug diclofenac [34,48].

Following marathon race participation, athletes experience changes of hemostasis associated with a pro-thrombotic state [20]. Two studies investigated the effect of bromelain on platelet aggregation. In vitro bromelain proteases reduced human platelet aggregation [49]. In rats Livio et al. also demonstrated reduced platelet aggregation and fibrinolytic effects after administration of bromelain [50]. Orally applied bromelain showed an inhibition of thrombus formation in rat mesenteric vessels [49].

In summary, ingestion of oral hydrolytic enzymes and flavonoids is hypothesized to function as a countermeasure strategy to marathon-induced inflammation, muscle damage and soreness, negative changes in rheological status, and increased URTI incidence. These negative physiologic effects after marathon competition prolong recovery. For athletes it is very important that the regeneration is as effective and as short as possible. A product that could support recovery from strenuous exercise would be beneficial not only for high-performance athletes but also for non-elite athletes.

## Abbreviations

ANOVA: Analysis of variance; CDISC: Clinical data interchange standards consortium; CK: Creatine kinase; (hs-)CRP: (high-sensitivity) c-reactive protein; eCRF: Electronic case report form; GCP: Good clinical practice; GOT: Glutamate oxalacetate transaminase; IL: Interleukine; IMP: Investigational medical product; LDH: Lactate dehydrogenase; SPSS: Statistic package for social sciences, statistical program; SAS: Statistical data format; SAE: Serious adverse event; tPA: Tissue plasminogen activator; URTI: Upper respiratory tract infections.

## Competing interests

None of the authors had any personal or financial conflicts of interest.

Funding for the study was partly received by MUCOS Pharma GmbH & Co. KG, Berlin. The funders had no direct role in the study’s design, conduct, analysis, interpretation of data and reporting beyond approval of the scientific protocol in peer review for funding. No other grants were received.

## Authors’ contributions

All authors made substantial contributions on the design of the trial. VG wrote the first and final draft manuscript and contributed to the conception of the final draft of the trial protocol and was responsible for the trial conduct and acquisition of data. As principal investigator JS wrote the first and final draft of the trial protocol. JS supervised the conduction of the trial and coordinated the cooperation between different parties. All other authors provided parts of the manuscript and revised the manuscript critically for important intellectual content and approved the final version to be published.

## Pre-publication history

The pre-publication history for this paper can be accessed here:

http://www.biomedcentral.com/2052-1847/6/8/prepub
